# A Booklet on Participants’ Rights to Improve Consent for Clinical Research: A Randomized Trial

**DOI:** 10.1371/journal.pone.0047023

**Published:** 2012-10-19

**Authors:** Jocelyne R. Benatar, John Mortimer, Matthew Stretton, Ralph A. H. Stewart

**Affiliations:** Green Lane Cardiovascular Service, Auckland City Hospital, University of Auckland, Auckland, New Zealand; The George Washington University Medical Center, United States of America

## Abstract

**Objective:**

Information on the rights of subjects in clinical trials has become increasingly complex and difficult to understand. This study evaluates whether a simple booklet which is relevant to all research studies improves the understanding of rights needed for subjects to provide informed consent.

**Methods:**

21 currently used informed consent forms (ICF) from international clinical trials were separated into information related to the specific research study, and general information on participants’ rights. A booklet designed to provide information on participants’ rights which used simple language was developed to replace this information in current ICF’s Readability of each component of ICF’s and the booklet was then assessed using the Flesch-Kincaid Reading ease score (FK). To further evaluate the booklet 282 hospital inpatients were randomised to one of three ways to present research information; a standard ICF, the booklet combined with a short ICF, or the booklet combined with a simplified ICF. Comprehension of information related to the research proposal and to participant’s rights was assessed by questionnaire.

**Results:**

Information related to participants’ rights contributed an average of 44% of the words in standard ICFs, and was harder to read than information describing the clinical trial (FK 25 versus (vs.) 41 respectively, p = 0.0003). The booklet reduced the number of words and improved FK from 25 to 42. The simplified ICF had a slightly higher FK score than the standard ICF (50 vs. 42). Comprehension assessed in inpatients was better for the booklet and short ICF 62%, (95% confidence interval (CI) 56 to 67) correct, or simplified ICF 62% (CI 58 to 68) correct compared to 52%, (CI 47 to 57) correct for the standard ICF, p = 0.009. This was due to better understanding of questions on rights (62% vs. 49% correct, p = 0.0008). Comprehension of study related information was similar for the simplified and standard ICF (60% vs. 64% correct, p = 0.68).

**Conclusions:**

A booklet provides a simple consistent approach to providing information on participant rights which is relevant to all research studies, and improves comprehension of patients who typically participate in clinical trials.

## Introduction

Informed consent is essential to protecting the participant in medical research. [1]–[4] It has two components: a verbal component, which consists of a discussion, usually between the potential participant and the investigator, and a written component, which consists of documents presented to the potential participant, and is intended to facilitate discussion with the potential participant. Despite the potential for the written component to enhance the process, numerous problems have been identified with informed consent documents. [5], [6] The language used has become unnecessarily legalistic and information on participants’ rights has increased disproportionally. [7] These changes reflect the perceived need for ‘full disclosure’ [8] which has expanded to include information on who has access to anonymous data and potential conflicts of interest for parties not involved with the medical care of the participant. The result is that written informed consent forms (ICFs) have become increasingly difficult for participants to read and understand. [9]–[16] Many potential participants do not read the information they are given, and those who try may fail to understand what they are reading [6], [10]–[12].

Comprehension of informed consent information is even more difficult for individuals who have poor literacy as well as patients who are unwell at the time of consent. [14] Patients who do not fully understand the implications of participation in a research trial are more likely to regret their decision and to withdraw from the study later. [17] Previous approaches suggested to address these problems have had only modest success. [18].

The assessment process undertaken by Institutional Review Boards (IRBs) may worsen rather than improve these problems. In an evaluation of ICFs for clinical trials assessed by IRBs in Veteran’s Administration Medical Centers, most changes made to the ICFs increased their length and made them harder to read. [19] For large clinical trials this process may be repeated across tens or hundreds of sites.

One possible solution is to use a simple, easy to read booklet on participants’ rights, which is sensitive to the culture and law of the country, and is relevant to most clinical research studies. This booklet could be approved by the IRB and regulatory bodies for use across all studies together with a much shorter study- specific ICF. The primary aim of this study is to investigate whether this approach improves understanding by potential research participants. In addition we investigate whether simplifying the trial specific ICF with simpler language, diagrams of study design, and tables of expected visits, improves patient understanding of a clinical trial.

## Methods

In this study two approaches were taken to assess how easy different component of ICFs are to comprehend. First documents were evaluated using the Flesch-Kincaid (FK) reading ease and grade level scores. [20], [21] Second, hospital inpatients were randomly assigned to one of three ways of providing written informed consent information; a standard ICF (control arm), a short ICF + booklet, or a simplified ICF + booklet. Recall and comprehension were then evaluated using a questionnaire.

### Review of ICFs and Development of Study Materials

Informed Consent Forms (ICFs) from 21 ethically approved contemporary international clinical trials undertaken at our institution were evaluated for length and readability. Content was divided into study- specific information and general information about participants’ rights. The word count and page length were determined for each, and readability assessed using the FK Reading ease and grade level scores. [20], [21] These scores use algorithms based on word and sentence length to evaluate the comprehension difficulty of a document. The FK reading ease score is inversely related to how comprehensible the document is; legal documents typically score 10 and comics 90. The grade level score correlates with the number of years of education generally required to understand the text.

To ensure that the ability to shorten and simplify ICFs was feasible across a range of studies, and that the result of the evaluation could be applied to different study designs, three representative ICFs were selected from the audit as templates for the randomised evaluation. The selected ICFs most closely matched the average length and readability score from the audit and included different types of studies and had different sponsors. Three ICFs were used to ensure that the ability to shorten and simplify ICFs is feasible across a range of studies with different study designs. After changing propriety and drug names to fictional alternatives they were either left unchanged (standard ICF), had general information on participants’ rights removed (short ICF) or were further modified by using simpler language and shorter sentences including a study diagram and a table of study visits (simplified ICF). Both the short and simplified ICF were given to participants with the booklet.

A booklet to inform participants about their rights and responsibilities was developed after review of ICFs in current use, Good Clinical Practice guidelines, [3] Food and Drug Administration regulations [1] and the European Union directive. [22] The process used to develop and validate the booklet has been described separately [23].

A questionnaire was designed to evaluate comprehension based on a previous study on informed consent. [15] Recall of the study information was assessed with 8 multiple choice questions related to the specific study and 5 on participant’s rights. The questions relating to rights assessed recall of the right to confidentiality and who has the right to access source data, that participation is voluntary with the right to withdraw, compensation should injury occur, and what happens to blood samples collected during the study (of particular relevance to Maori). In a pilot evaluation two questions from each were more discriminatory. These questions scored 2 points for a correct response and 1 point for a partly correct response. Other questions had 1 correct answer which scored 1 point. The number of correct answers out of 17 was expressed as a percentage. A second section related to the participants’ subjective experience of the process, with questions on how much of the information they read, how much they understood, and the time spent reading the information. The format of the 3 questionnaires was the same with minor changes made to the study specific questions, to make them relevant to the corresponding study design.

### Procedures

Ethics approval was granted by the Northern X ethics committee, New Zealand. Ethics number NTX/10/EXP206. On chosen study days between November 2011 and January 2012 all hospital inpatients from 8 wards of various specialties were screened for participation in the study. After review of hospital charts individuals were excluded if they were aged less than 18 years, unable to read English, they were incapacitated, or expected to be discharged within 3 hours. All others were approached to participate in the study.

The ethics committee ruled that written informed consent for this study was not needed because the study was low risk and an informed consent process could interfere with study results. Participants were told that we were assessing informed consent in the hospital. We asked if they would be willing to read an ICF, but gave no indication that a questionnaire assessing recall and comprehension would be administered. Participants who gave verbal agreement to read the documents were allocated a sequential study number and then randomly assigned to one of three possible arms from an opaque envelope; a standard ICF (control arm), a short ICF + booklet, or a simplified ICF + booklet. For each an ICF from 1 of 3 different studies was randomly allocated. Participants’ ethnicity, educational level, English as a first language, age and gender were documented. They were then asked to read the ICFs and booklet. Study staff returned 3–24 hours later, when the ICF’s and booklets were retrieved and a written questionnaire administered to participants who were medically stable. Subjects were given up to 2 hours to complete this with no assistance from others. Those that did not complete the questionnaire were considered ‘non-completers’ and the reason recorded. Screening was stopped when the sample size was reached from the pre-specified power calculation.

### Statistical Analysis

The primary objective was to determine whether the booklet and short or simplified ICF improved the proportion of correct responses to a questionnaire which assessed comprehension and recall of informed consent when compared to a standard ICF. Based on a pilot study we estimated that 60 completed questionnaires in each group would have 80% power (P<0.05) to detect a 15% difference between groups. Assuming one third of subjects would not complete the questionnaire a sample size of 282 was chosen. Baseline characteristics and scores were compared across the three arms using the analysis of variance (ANOVA). If significant differences were found, Tukey’s Honestly Significant Difference (HSD) multiple comparison procedure was used to assess the difference between each pair of randomized groups. Statistical analyses were performed with SAS software version 9.3 (SAS Institute, Cary, NC). All p-values resulted from two sided tests and a p-value of <0.05 was considered statistically significant.

The regional ethics committee approval number is NTX/10/EXP206.

## Results

### Review of Currently Approved ICFs

The length and reading scores for 21 currently approved ICFs, the 3 standard ICFs, 3 short and 3 simplified ICFs used for this study, as well as the booklet on participants’ rights are presented in Table 1. Currently approved ICFs were on average 18 pages long with over ∼4000 words. Information related to participants’ rights contributed an average of 44% of the words in currently used ICFs, and this information was harder to read than information describing the clinical trial (Flesch-Kincaid reading ease score 25 vs. 41, p = 0.0003). Both the simplified ICF and the information on participant rights in the booklet were easier to read and used fewer words (Table 1).

**Table 1:** Comparison of length and reading ease of information provided in informed consent forms.

	Currently used ICFs Median (IQR)(n = 21)	Standard ICF Median (range)(n = 3)	Short ICF Median (range)(n = 3)	Simplified ICF Median (range)(n = 3)
**Study specific information**				
Number of pages	10 (8–11)	8 (6–11)	8 (6–11)	5(4.5–5.5)
Word count	4392 (3058–5676)	4674 (3110–5623)	4674(3110–5623)	1651(1161–2354)
Flesch-Kincaid Grade level*	13 (11–14)	13 (11–14)	13 (11–14)	10 (9–10)
Flesch-Kincaid Reading Ease#	41 (35–45)	42 (34–50)	42 (34–50)	50 (47–53)
**Participants’ rights and general research information**			**Booklet**	**Booklet**
Number of pages	8 (6–10)	8 (5–11)	8	8
Word Count	3449 (2550–4789)	3450 (2568–4786)	1423	1423
Flesch-Kincaid Grade level*	16 (15–16)	16 (15–18)	11	11
Flesch-Kincaid Reading Ease#	25 (19–35)	27 (19–31)	42	42
**TOTAL**				
Number of pages	18 (14–21)	16 (11–22)	16 (14–19)	13(12–14)
Word Count	7841 (5608–10465)	8124 (5678–10049)	6097 (4533–7046)	3074 (2584–3777)
Flesch-Kincaid Grade level*	14.8 (13–16)	14.8 (13–17)	12 (11–13)	10 (9–11)
Flesch-Kincaid Reading Ease#	33 (27–40)	35 (30–37)	42 (36–48)	46 (44–48)

Results presented are median and range.

* Flesch-Kincaid Grade level. The score relates to the grade level required to read the document e.g. a level of 12 indicates that the participant needs to be in grade 12 (the highest secondary school year) to read the document.

# The Flesch-Kincaid Reading Ease score: Higher indicates easier to read (comics typically have a score of 90 and legal documents 10).

### Randomized Study

**Figure 1:**
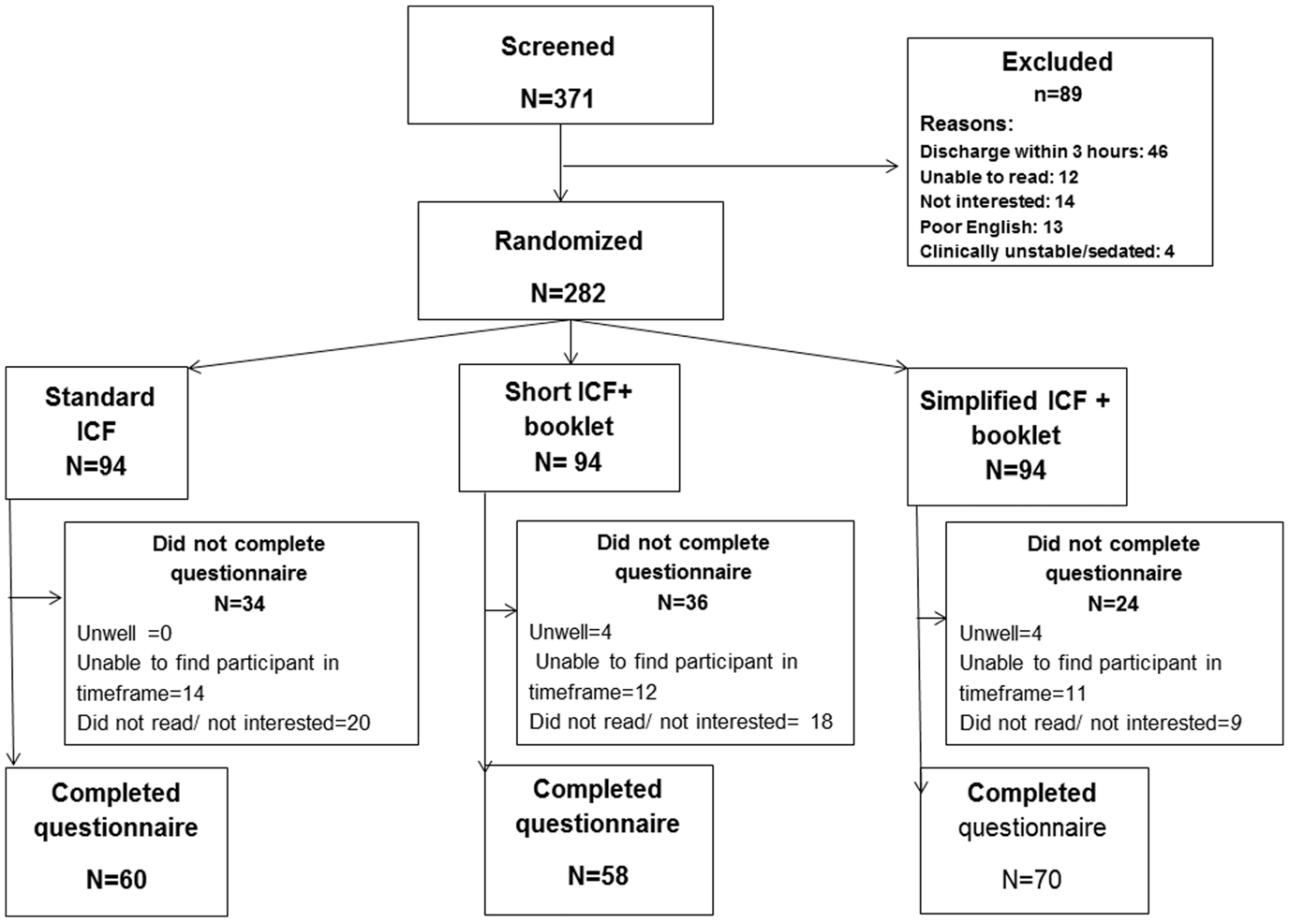
Study schematic.

Of the 371 patients screened for the study 282 were randomized (Figure 1). Reasons for exclusions of 89 subjects were expected to be discharged within 3 hours (n = 46), unable to read (n = 12), did not understand English (n = 13) and clinically unstable (n = 4). 14 patients declined participation because they were not interested in the study, mainly as they felt overwhelmed or unwell. Baseline demographic data is presented in Table 2. The average age was 63 years and 61% were male, 29% European, 56% New Zealanders, 8% Maori, 5% Pacific Islanders and 2% Asian. There were no significant differences in any demographic characteristic between randomized groups.

**Table 2:** Description of study population with each group divided in those who completed questionnaire (completers) and those who did not complete questionnaires (non-completers).

Randomized	Standard ICF N = 94		Short ICF + booklet N = 94		Simplified ICF + booklet N = 94	
	Completers	Non-completers	Completers	Non-completers	Completers	Non-completers
Number	60 (64%)	34 (36%)	58 (62%)	36 (38%)	70 (75%)	24 (25%)
Age, years (SD)	63 (16)	68 (13)	62 (15)	67 (16)	63 (16)	71 (14)
Gender (male) %	61	68	65	61	62	70
**English as first language**	52 (87%)	30 (88%)	54 (93%)	33 (93%)	64 (91%)	22 (93%)
**Ethnicity**						
Maori	3 (5%)	8 (23%)	3 (5%)	7 (22%)	4 (6%)	5 (21%)
New Zealander	50 (84%)	25 (76%)	48 (83%)	28 (77%)	58 (83%)	19 (78%)
Pacific Islander	5 (9%)	1 (3%)	5 (9%)	1 (3%)	4 (7%)	1 (3%)
Asian	2 (3%)	0	2 (3%)	0	2 (4%)	0
**Highest educational Level n (%)**						
Primary School (up to age 13)	3 (5%)	2 (6%)	3 (5%)	2 (5%)	2(3%)	1 (4%)
Secondary School (up to age 18)	30 (50%)	17 (50%)	31 (53%)	19 (53%)	37(53%)	13 (54%)
Tertiary (University or Technical)	27 (45%)	14 (41%)	24 (42%)	14 (39%)	31(44%)	9 (38%)
Unknown	0	1 (3%)	0	1 (3%)	0	1 (4%)

Data is from responses to demographic questions in the questionnaire on randomization.

Older participants (66 years, ±16 vs. 61 years ±16, p = 0.02) and Maori (66% vs. 29%, p<0.001) were less likely to complete the questionnaire, however there were no differences between randomized groups. The total completion rate was higher for the simplified ICF + booklet (75%) compared to the standard ICF’s (64%, p = 0.05) and the short ICF + booklet (62%, p = 0.04).

188 (67%) of the 282 randomized subjects completed the study questionnaire. Of these subjects 42% had attended secondary school and 51% a university or other tertiary institution. The completion rate was higher for the simplified ICF + booklet (74%) compared to the standard ICF’s (64%, p = 0.05) and the short ICF + booklet (61%, p = 0.04). Non-completers were on average older than completers (66±16 vs. 61±16 years, p = 0.02), but there was no significant difference by sex or educational level (Table 2). Maori were more than twice as likely to not complete the questionnaire compared to other ethnic groups combined (67% vs. 29%, p = <0.001).

For questions related to participants’ rights, there were fewer correct responses for the standard ICF (49%, CI 43–55), compared to the short ICF + booklet (64%, CI 60–66, p = 0.0003) or the simplified ICF + booklet (61%, CI 59–63, p = 0.02). The most frequently correct response was to the right to withdraw from the study and that the study was voluntary for the standard ICF (70%), short ICF + booklet (86%) and simplified ICF + booklet (89%). The question that had the lowest correct response rate for the standard ICF (29%), short ICF + booklet (40%) and simplified ICF + booklet (40%) related to confidentiality and who would have access to source data. In addition participants in this group were more likely to report reading all the information, and to feel they understood the information (Table 3).

**Table 3:** Responses to the questionnaire.

	Standard ICF	Short ICF + booklet	Simplified ICF + booklet	Booklet + Short ICF or Simplified ICF vs Standard ICF	Short ICF + booklet vs Simplified ICF + booklet
	n = 94	n = 94	n = 94	p value	p value
**Responses to written questionnaire evaluating comprehension and recall**					
Number completing questionnaire, n (%)	60 (64 )	58 (61 )	70 (74)	0.68	0.04
Information about study, % correct answers	55 (49–60)	60 (54–67)	64 (57–70)	0.05	0.68
Participants’ rights, % correct answers	49 (45–53)	64 (60–67)**	61 (57–67)*	0.0008	0.73
Total, % correct answers	52 (47–57)	62 (56–67)*	62 (58–68)*	0.009	0.97
**Participant feedback on consent process**					
How well did you understand the information? not at all = 0……4 very well	2.5 (1.8–3.1)	2.9 (2.2–3.7)*	2.7 (2.1–3.5)*	0.009	0.47
How reassured are you that all concerns have beenaddressed? not at all = 0…….4 very reassured	2.9 (1.9–3.4)	2.9 (2.1–3.8)	3.0 (2.1–3.9)	0.53	0.57
How much of the information about the study didyou read? %	79 (50–87)	85 (65–100)	87 (70–100)	0.03	0.97
How much of the information about your rights didyou read? %	75 (50–84)	87 (70–100) *	84 (65–100)*	0.05	0.17
How long did you spend reading the ICF? time in minutes	22 (18–30)	19 (16–25)	17 (15–22)	0.05	0.78
Would you participate in this study if invited? n (%) yes	27 (44)	22 (37)	37 (59)*	0.2	0.04

Results are number and % =  n (%) or mean (95%confidence interval).

* P<0.05, **p<0.005 compared to standard ICF.

For questions related to the study, there were fewer correct responses for the standard ICF (55%, CI 49–60) compared to the short ICF + booklet (60%, CI 54–67, p = 0.4) and the simplified ICF + booklet (64%, CI 57–70, p = 0.08), but these differences were not statistically significant (Table 3). No difference in correct responses was seen between the three template ICFs, for the standard ICF (54% vs. 53% vs. 54%, p = 0.98), the short ICF + booklet (60% vs. 60% vs.62%, p = 0.79) and simplified ICF+ booklet (63%, 65%, 63%, p = 0.85).

The proportion reporting reading all the information and feeling they understood the information, were similar. However, more participants given the simplified ICF + booklet indicated they would participate in the study if invited (59% vs. 37%, p = 0.04). The standard ICF took longer to read than the short ICF + booklet and the simplified ICF + booklet.

## Discussion

The ICFs evaluated in this study are similar to those used for large clinical trials in most countries, [9] but their length and complexity conflicts with the core principle that information on research should be easy for potential participants to understand. Suggested approaches to improve this problem include the use of multi-media, diagrams and introductory pages to complement or explain the ICF. [18] However these solutions do not reduce the large amount of information individuals need to consider and have had only modest success. [18] They also do not address the increasing use of legalistic language, especially in those sections detailing participants’ rights. The use of this language is likely to be confusing and may appear to waive participants’ rights, in direct contravention of Good Clinical Practice guidelines. [3] Participants frequently misconstrue the purpose of informed consent documents, perceiving them to be for the protection of the investigators, rather than for their own benefit [6].

Information related to the rights of participants contributed nearly half of the total information included in currently used ICFs at our hospital, and this information was harder to read than the description of the proposed study. Separating the general information on rights from that related to the aims and procedures of the research study, and providing this information in a booklet in a simple way, improved patient comprehension.

Using a booklet to present information on rights for all studies has many potential advantages. Removing legalistic language reduces the misconception that the ICF mitigates risk. Legal rights can be made more reflective of the country’s legal system, and when appropriate indigenous rights can be included. The booklet can be translated into different languages while maintaining consistency in the information included. A standard booklet would also simplify the submission and review process for ethics committees, because the information on participants’ rights has already been agreed, so the focus can be on issues related to the proposed research study. This is particularly relevant given the increasing workload for many ethics committees, and evidence that interventions by ethics committees are often inconsistent and do not improve ICF’s [19].

Simplifying the study-specific information had less impact on recall of the information related to the proposed clinical study. Removing legalistic language appears to be the most effective intervention. There are, however, other advantages to simplifying the ICF. Participants presented with simpler information were more likely to read the ICF and to complete the questionnaire, and may be more engaged when presented with simpler information. They also spent less time reading the ICF without impairing comprehension. These advantages may be particularly beneficial in situations when the time available for consent is short, such as in the emergency setting [15].

### Study Limitations and Strengths

#### Strengths

This study evaluated understanding and recall based on the information given to participants typically recruited for clinical studies, including older and acutely unwell in-patients undergoing active investigation and treatment. Three currently used ICFs with different study designs were used as templates to assess whether this approach could be used across a range of studies. The booklet was developed after feedback from a broad group of people with an interest in informed consent, [15] and a pilot study was used to refine the study design, questionnaire and booklet.

Both objective measures of recall and perceptions of the process, including reasons for not completing the questionnaire were assessed. Subjective measures are important even if harder to quantify and interpret, because they may influence the decision to participate in a study.

The use of a separate booklet to inform subjects of their rights should be easy to implement. In New Zealand the National Ethics Advisory Committee’s, whose statutory functions include determining nationally consistent ethical standards for research, has recommended the booklet and template ICF are available for use by investigators on the ethics committee website.

### Limitations

In this study the verbal component of the consent process was excluded to allow evaluation of the written information alone. Overall levels of comprehension and recall were relatively poor, even when every effort was made to use simple language and avoid unnecessary information. This finding is consistent with previous research, [18] and indicates that providing simple clearly written information is not sufficient to ensure that consent is informed. Discussion is therefore crucial to answer questions participants have, and to ensure that consent is both informed and voluntary. An advantage of the booklet is that it provides information that can be more easily understood by the participant or their advocates when questions arise later. However a booklet will not work for illiterate populations. Patients from some ethnic groups, including Maori in this study, may be less engaged if only written information is provided. [24] A verbal discussion which includes family and other individuals who are sensitive to the culture would be needed to obtain quality consent in these settings.

The Flesch-Kincaid test uses algorithms for numbers of words per sentence and syllables per word to assess the reading ease and grade level of documents, but does not directly determine how comprehensible the document is. In this study improvements in comprehension and recall between ICFs were consistent with differences in the Flesch-Kincaid test scores.

More subjects randomized to the simplified ICF completed the questionnaire, and for this arm it is possible a modest difference in completion rate biased evaluation of comprehension. Subjects randomized to the simplified ICF were also more likely to indicate they would participate in the clinical trial if eligible. Greater willingness to participate in studies with simpler information has been reported by others [13] and may not necessarily relate to comprehension of the study.

In this study subjects were not consenting for a clinical trial designed to evaluate a therapeutic intervention. Participants in this study were however typical of those invited into clinical trials. Further studies assessing a standard ICF and simplified ICF + booklet as part of a real clinical trial would be valuable.

### Data Sharing

The anonymised dataset is available from Jbenatar@adhb.govt.nz. Consent was not obtained but the presented data are anonymised and risk of identification is low.

### Conclusion

The written information in informed consent forms provides a framework to facilitate the conversation between investigator and participant which is crucial to the informed consent process. Providing information on the rights of research participants in a separate booklet helps to ensure this information is consistent and clear, and improves understanding. A separate booklet may also simplify processes for IRB’s, allowing them to focus predominantly on the specific research proposal.

## Supporting Information

Appendix 1 **Booklet “Participant Information for Clinical Studies”.**
(DOCX)

Appendix 2 **Study-Specific Informed Consent Form Template.**
(DOC)

Appendix 3- **Consort Checklist.**
(DOC)

Appendix 4- **Study Protocol.**
(DOC)
