# Supplementary material for: A Booklet on Participants’ Rights to Improve Consent for Clinical Research: A Randomized Trial
Source: PLoS One. 2012 Oct 19;7(10):e47023. doi: 10.1371/journal.pone.0047023 (PMC3477160; doi:10.1371/journal.pone.0047023)
Supplement: Appendix 1 — Booklet “Participant Information for Clinical Studies”. (DOCX) [file pone.0047023.s001.docx]

Participant Information for Clinical Studies

**Important contacts**

Principal investigator: ………………….………….

Study coordinator: ……………………..…………..

**Independent advice services**

Māori health support: …………………….………..

Health and disability advocate:

Free phone: 0800 555 050

Free fax: 0800 2787 7678

Email: advocacy@hdc.org.nz

Website: advocacy.hdc.org.nz

Health and disability advocacy is a free service provided under the Health and Disability Commissioner Act.

**If you need an INTERPRETER, please tell us.**

**Introduction**

This booklet provides information about your rights and welfare in a clinical study. Please read it carefully.

You also need to carefully read the separate **Participant Information and Consent Form**.

You will find definitions for some commonly used words at the end of this booklet.

You are welcome to ask your friends, family and whanau to help you understand this information.

**Participation in all studies is voluntary.**

**It is important that all of your questions are answered before you decide to participate in the study.**

**Why are clinical studies done?**

Studies are done to determine which treatments are beneficial, and whether or not they are harmful.

**Why do people participate in clinical studies?**

Medical research benefits other people. Sometimes it benefits study participants.

**Do I have to take part in the study?**

No, participation is **voluntary**.

You can withdraw from the study at any time.

If you decide not to take part, or to withdraw, you will continue to receive the healthcare to which you are entitled.

**How will participating in the study affect my healthcare?**

Your usual doctor (the specialist who you have been seeing or your general practitioner) will continue to care for you during the study. If you agree we will tell them that you are participating in the study.

New medical information about you may be found during the study. If this information is important to your health, your usual doctor will be informed and will discuss it with you further.

You will also be told of any new information that may affect your willingness to take part in the study.

**What will I have to do during the study?**

For the study to be successful, the researchers will need your commitment.

It is important that you attend all of the required appointments. You will also be asked to do certain things. For example, you may be asked to take medication, or to report symptoms.

*Refer to the Participant Information and Consent Form for further information about what you will be asked to do during the study, or ask the investigator*.

**Blood and tissue samples**

Studies often require blood and/or tissue samples to be taken and stored.

If blood and/or tissue samples are needed for the study:

- samples may be sent overseas
- samples may be stored for future testing (however, ethics committee approval is required for all testing)
- you can ask for your samples to be destroyed and to be notified when this been done (usually, samples cannot be returned)
- **genetic testing** will only be done on your samples if you sign a specific consent form

*Please read the Participant Information and Consent Form to find out what will be needed for the study, or ask the investigator.*

**What do I need to tell the researchers?**

You must tell the researchers

- Medications you are taking (including over-the-counter and herbal medications).
- if you have any health concerns
- if you are pregnant, or become pregnant
- if you are currently involved, or become involved, in another study
- if you decide you want to withdraw from the study, or stop the study treatment
- how you can be contacted in the future if it becomes necessary to do so

**Will I be paid?**

People are not usually paid for participating in clinical studies.

Costs related to your participation in the study may be reimbursed.

The study treatment will be made available to you at no cost.

*Refer to the Participant Information and Consent Form for further information about payment and reimbursement for the study, or ask the investigator*

**Confidentiality**

All personal information about you will be kept secure and private.

No material that could identify you will be used in any reports.

The investigator, the other researchers involved in the study, and representatives from the sponsor will be able to see your medical records.

Certain organisations may access your medical records to check that the study information is accurate as required by law. These include:

- independent ethics committees
- inspectors from New Zealand government regulatory agencies
- inspectors from overseas government regulatory agencies

You may ask to see your personal information and correct it if necessary.

**Why would the study treatment be stopped early?**

The study treatment will be stopped if:

- the investigator thinks it is in your best interests (for example, if you get new health problems during the study that would affect your participation, or if tests show that you should not be on the study treatment)
- you become pregnant, or decide you want to become pregnant (if the study treatment is not known to be safe during pregnancy)
- you do not follow instructions for treatment, or do not attend all of the required appointments
- the sponsor decides to withdraw the study

**What will happen when the study has ended?**

You will continue to see your usual doctor for treatment and care.

You will be able to see the medical information that has been collected about you during the study.

The study treatment may be made available to you at the end of the study, but this will depend on whether the treatment is registered in New Zealand, and whether the study sponsor agrees to supply it.

The investigator will be able to tell you the results of the study. However, this may take some time.

**Who approves studies in New Zealand?**

All clinical studies are approved by an ethics committee accredited by the Health Research Council. The ethics committee is made up of lay and professional people who review each study to ensure that your rights and welfare are protected.

Scientific committees also review and approve studies, on behalf of the Director-General of Health.

**Injury and compensation**

The investigator and sponsor will ensure you receive appropriate healthcare if you are injured as a direct result of your participation in the study. You may also be able to claim compensation.

Compensation will be provided by either the Accident Compensation Corporation (ACC) or the sponsor, depending on the study.

Injury caused by an investigator may be covered by the investigator’s liability insurance if it is not compensated for by ACC or the sponsor.

*Refer to the Participant Information and Consent Form to find out which of these would apply to the study, or ask the investigator.*

**ACC**

If you are injured as a direct result of your participation in the study you may be covered by ACC.

You will have to lodge a claim with ACC, which may take some time to assess.

If your claim is accepted, you will receive funding to assist in your recovery. ACC contributes to treatment and other costs, but this might not cover all of your expenses. ACC does not compensate for mental injury unless it is the result of a physical injury they accept.

Lodging a claim with ACC may affect your right to sue the investigator, sponsor, and hospital.

For more information about ACC, visit www.acc.co.nz.

**Sponsor**

If the study is being done mainly for the benefit of a sponsor, you will not be covered by ACC.

If you are injured as a *direct* result of your participation in such a study, the sponsor is responsible for compensation.

If your injury is the result of a failure by the investigator (because he or she has deviated from the proposed plan of research, for example) you will receive compensation from the investigator and hospital.

The compensation you receive will depend on the type and severity of your injury.

**Important information you should know if you identify as Māori (translated version to)**

All research in New Zealand follows the articles of the Treaty of Waitangi.

All studies go through an ethics review process, which includes consultation with a Māori representative.

Blood and/or tissue samples may be taken during the study, and these may be stored or sent overseas. Some iwi disagree with the storage and sending of samples overseas, out of respect for whakapapa. Your iwi may advise you to consult with them prior to participating in a study.

You might like to discuss the possibility of your participation in the study with your whanau, and let them know if you do decide to participate.

**You have the right to choose whether or not to participate in the study.**

**Please discuss any questions or concerns you have with the investigator.**

*Thank you for reading this information.*

**Commonly used words**

Clinical study: Any study involving human participants that is designed to assess the effects of a treatment. These include controlled trials and observational studies.

Controlled trial: A study in which some participants receive the study treatment, while others (those in the control group) receive either a placebo, standard treatment, or no treatment.

Observational study: A study in which the researchers simply observe behaviour without influencing or interfering with it.

Double blinded study: A study in which neither the participant nor the researchers know whether the participant is receiving the treatment or a placebo. However, in an emergency the researchers can find out what treatment the participant is on.

Single blinded study: A study in which the participant does not know whether they are receiving the treatment or a placebo. However, the researchers know.

Randomization: A process that assigns participants by chance to either the treatment group or the control group of a study, to avoid any selection bias.

Placebo: A treatment that has no therapeutic effect (for example, a pill that contains no active medication).

Investigator: A researcher who carries out a clinical study.

Sponsor: An organisation that designs, manages, and/or finances a clinical study.

Independent data-monitoring committee (IDMC): A committee that checks the progress and safety of a clinical study. It tells the sponsor whether to continue, change, or stop a study and ensures that the ethics committee that approved the study is kept informed about adverse events.

Accident Compensation Corporation (ACC): An organisation that provides no-fault injury cover for all New Zealand residents and overseas visitors. The Accident Compensation Corporation is governed by the Accident Compensation Act 2001.

**Phases of studies**

Phase One: ‘First in man’ studies. These look mainly at the side effects associated with increasing doses of the drug. They may give early information on whether or not the treatment works.

Phase Two: These studies look at whether or not the treatment works, and at common short-term side effects and risks.

Phase Three: These studies are bigger than phase one and two studies. They look at how well the treatment works, and at what the long-term side effects are. These studies are done before the drug is put on the market.

Phase Four: These studies are done on treatments that are already on the market. They look at additional benefits of the treatment, and at its risks.

*Refer to the Participant Information and Consent Form to find out which phase the study is in, or ask the investigator.*

**Useful websites**

Health and Disability Commissioner: [www.hdc.org.nz](http://www.hdc.org.nz)

Health Research Council: [www.hrc.govt.nz](http://www.hrc.govt.nz)

Ministry of Health: [www.health.govt.nz](http://www.health.govt.nz)

The booklet has been approved by the New Zealand Heath and Disability Commission and by the national ethics committees.
