# Supplementary material for: A Booklet on Participants’ Rights to Improve Consent for Clinical Research: A Randomized Trial
Source: PLoS One. 2012 Oct 19;7(10):e47023. doi: 10.1371/journal.pone.0047023 (PMC3477160; doi:10.1371/journal.pone.0047023)
Supplement: Appendix 2 — Study-Specific Informed Consent Form Template. (DOC) [file pone.0047023.s002.doc]

LETTERHEAD TO BE INSERTED

Instructions for investigators:

Use simple easily understood language

do not duplicate the information in the booklet “**Participant Information for Clinical Studies***”*

Please keep the ICF to 4-5 pages maximum- try use diagram and visit flow chart

Please keep the information sheet relevant- it is not a legal document

delete instructions in grey

| **CONSENT TO TAKE PART IN A RESEARCH STUDY** | |
| --- | --- |
| **TITLE:** |  |
| **PROTOCOL NO.:** |  |
| **SPONSOR:** |  |
| **INVESTIGATOR:** |  |
| **Institution    Telephone** |  |

Brief Introduction

We would like to invite you to take part in a research study called the **XXX** study. If you have any questions please feel free to ask the investigator at any time. **Please read the supplementary booklet called “Participant Information for Clinical Studies”**

Purpose of the study

**EXAMPLE** :We are inviting you to join this study because you have had a heart attack. Heart attacks are caused by narrowing of the arteries (atherosclerosis) and DD is thought to be involved in making arteries narrow.

This trial is looking at a new treatment called AA. It works by lowering DD. We hope that this can reduce the risk of heart or stroke even more when taken with all other medication. This study is to make sure that AA reduces risk without causing harm. The way we do this is by comparing what happens to people who are given AA compared to people given a look alike inactive tablet (placebo). Your normal medication is not stopped; the study drug is given as an extra tablet.

Where will the study be held?

You will be seen at EE clinics at ** hospital

About the study

**EXAMPLE**: About XXX patients will be enrolled in this study in YY countries. We hope to enroll ZZZ patients at this hospital

You will be in the study for over 3 years. The length of the study may be shorter or longer based on the recommendation from the independent board that monitors the study called the Data Safety monitoring Committee (DSMC).

Study design (**diagram preferred)- For example**

**AAA** tablet Twice a day

**Placebo** (dummy look- alike tablet) twice a day

Randomisation

You are seen at clinic at 1 month, then every 3 months. We will also phone you between visits to see how you are. There are approximately 10 clinic visits over the 3 year study period.

End of study

30 day follow up visit by phone

50%

50%

What will happen during the study?

EXAMPLE: You will be issued a card to show you are in the trial. Please show this card whenever you see a doctor.

Please look at the flow chart which will give an idea about thestudy visits. When you see us at the clinic we will:

- Take full medical history and do a full examination at the first and last visit for the study.
- Take blood tests and measure your blood pressure, HR and weight at every visit.
- We will do an ECG (electrocardiogram) at some visits
- If you are a woman who could potentially fall pregnant, we will do pregnancy tests at the beginning and end of the study.
- We will provide breakfast and give you study medication to last until the next visit

What you will be asked to do

EXAMPLE

- You will be asked to take one tablet at the same time each day throughout the study.
- **Please do not crush the tablet**. Swallow the tablet whole
- If you miss your study drug one day, do not double up the dose, just skip the dose.
- Tell us of all prescription and non prescription medication that you are taking.
- Please bring back all used and unused bottles of study medication at each clinic visit
- Please do not drink more than 1 glass of grapefruit juice a day

If you stop taking the study drug, please tell us. You can still stay in the study and we will maintain contact with you. We can discuss whether you want to be contacted by phone or by clinic visits.

You can decide to stop the study completely and not be followed up anymore. We would prefer that you come to the clinic for a final study visit. At this visit you should return all unused drug.

Blood Samples

Your samples are sent to a central laboratory called BB Laboratories in CC laboratory. Some blood may be stored at the laboratory and be used for future tests. These tests will be related to this study and the study protocol. You can request that the stored bloods be destroyed.

Alternative therapy

If you decide not to take part in this study, the study doctor will be happy to explain the alternative treatments of heart disease to you.

Potential risks and discomforts

AAA has been tested in multiple studies involving both healthy people and people with heart disease. The most common side effects in people are an unusual smell of urine, faeces, or skin, change in taste and diarrhoea. Less common side effects are allergic reactions and

We may not know all the side effects of this medication. If you feel unwell when you are in the study please contact us. If you think you have problems because this medication, contact us.

Benefits

A benefit of being in this study is that you will be monitored frequently. We do not yet know if Vitex has beneficial effects on heart attacks and you may be on placebo.

Contraception and pregnancy

We do not know of the effects on AA on an unborn child. Please use adequate contraception during the study and for 1 month after stopping the study medication. Your study doctor will discuss this with you which methods are best and will do pregnancy tests at some visits.

**Your rights and responsibilities are described in the booklet “Participant Information for Clinical Studies”. Please read this and make sure all your questions are answered before you sign this consent form. Compensation is covered by RMI guidelines/ ACC (delete appropriately)**

***The study has been approved by the MMM ethics committee***

**Flow Chart of the study visits for XXX clinical study (example)**

This flow chart tells you what to generally expect each visit.. Please bring all study medication to each visit including all un-used study medication and empty bottles.

|  | **Randomization** | **Month1** | **Every 3 months** | **Yearly visits** | **Final visit** |
| --- | --- | --- | --- | --- | --- |
| **Expected duration** | **1 ½ hours** | **1 hour** | **¾ hour** | **1 hour** | **1 ½ hour** |
| **Study explained/ consent** | **√** |  |  |  |  |
| **Full medical history** | **√** |  |  |  |  |
| **Questions about health and medication** | **√** | **√** | **√** | **√** | **√** |
| **Physical exam** |  | **√** |  | **√** | **√** |
| **Blood tests** | **√** | **√** | **√** | **√** | **√** |
| **ECG** | **√** | **√** |  | **√** | **√** |
| **Bring used and unused study medication** |  |  | **√** | **√** | **√** |
| **Urine Sample** |  | **√** |  | **√** | **√** |
| **Please call us if you are unable to come to your appointment** | | | | | |

**Thank you for reading this information leaflet**

**CONSENT**

I have read and I understand this information sheet and the booklet on my rights. I have had the opportunity to discuss this study. I am satisfied with the answers I have been given and that I have had sufficient time to consider whether to participate in the study. **YES/NO**

I consent to blood/tissue samples being sent to the XXX Laboratory **YES/NO**

I consent to blood /tissue samples being destroyed at the end of the study. **YES/NO**

I wish to receive a copy of the results. **YES/NO**

I agree to my GP or other current provider being informed of my participation in this study/the results of my participation in this study. **YES/NO**

**I consent to take part in this study.**

| **Participant Name (Print)** |  | **Participant Signature** |  |
| --- | --- | --- | --- |
| **Date** |  |  |  |
|  |  |  |  |
| **Investigator (print)** |  | **Signature of Investigator** |  |
| **Date** |  |  |  |
