# Supplementary material for: A Booklet on Participants’ Rights to Improve Consent for Clinical Research: A Randomized Trial
Source: PLoS One. 2012 Oct 19;7(10):e47023. doi: 10.1371/journal.pone.0047023 (PMC3477160; doi:10.1371/journal.pone.0047023)
Supplement: Appendix 4- — Study Protocol. (DOC) [file pone.0047023.s004.doc]

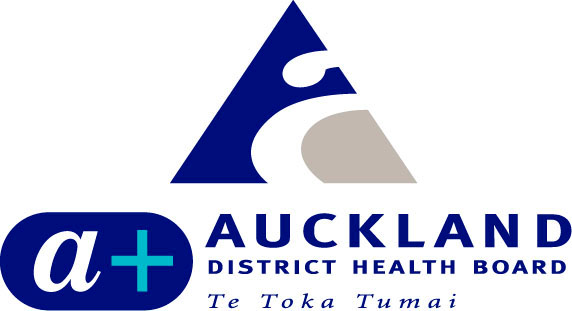


Randomised comparison of two approaches to providing patients with information on clinical trials

-The informed consent study

Investigators: Dr Jocelyne Benatar

Dr Ralph Stewart

Dr Samantha Jones

# Table of Contents

[Table of Contents 2](#__RefHeading___Toc293913470)

[Brief Summary 3](#__RefHeading___Toc293913471)

[Background to Proposed Research 4](#__RefHeading___Toc293913472)

[Aims of Research 6](#__RefHeading___Toc293913473)

[Aims 6](#__RefHeading___Toc293913474)

[Study hypotheses: 6](#__RefHeading___Toc293913475)

[The objective 6](#__RefHeading___Toc293913476)

[Research Design 8](#__RefHeading___Toc293913477)

[Evaluation of Booklet Validity 8](#__RefHeading___Toc293913478)

[Materials and Procedure 8](#__RefHeading___Toc293913479)

[Randomised Study to Evaluate Consent Process Using Short ICF and Handbook Vs. Standard Consent 8](#__RefHeading___Toc293913480)

[Participants and Sample Size Calculations 8](#__RefHeading___Toc293913481)

[Materials 9](#__RefHeading___Toc293913482)

[Procedure 10](#__RefHeading___Toc293913483)

[Analysis of Results 10](#__RefHeading___Toc293913484)

[Significance of study 10](#__RefHeading___Toc293913485)

[References 11](#__RefHeading___Toc293913486)

# Brief Summary

Informed consent must be obtained prior to participation in a clinical research trial. The informed consent form (ICF) is used to provide information to the patient and to demonstrate consent has been obtained. The ICF provides information specific to the trial (e.g. what the trial is testing, the number of visits required, etc) and general information about research studies, including risks, benefits, rights and obligations. The general information is applicable to all trials.

ICFs have become increasingly long and use more complex language. Readability tests suggest a tertiary level education is now needed to understand them. Previous studies suggest that ICF’s are often poorly understood. Length is also an issue, with those longer than 4 pages less likely to be read.

The aim of this research is to determine whether an easily understood booklet containing information relevant to any clinical research project, together with a shorter and simpler trial specific ICF, improves comprehension and recall compared to the standard long ICF. A randomised trial will assess comprehension and recall of a shorter ICF and booklet compared to a standard ICF. The content validity of the booklet will also be evaluated. Study participants will be cardiology inpatients similar to those invited to participate in clinical trials.

Results of the study are directly relevant to improving informed consent for clinical research at ADHB and in New Zealand.

# Background to Proposed Research

Informed consent is the process by which a person agrees to participate in a trial. This should be based on a clear understanding of the facts, implications, and future consequences of an action. ^1,2^ It implies the individual concerned understands the information presented and can make a reasoned decision based on this information on whether to participate in a clinical trial. Informed consent is designed to protect individuals participating in clinical research trials.

The informed consent form (ICF) is a tool used by the investigator to facilitate the process of obtaining consent and to demonstrate that the participant has consented to the study. The ICF is not a legal document. The ICF demonstrates that participants are informed of their right with all necessary components of consent. The investigator and the ethics committee decide on the wording of the ICF with sponsor input to ensure all risks related to the intervention included. ^1,3-5^The ICF includes trial specific information as well as more generic information relevant to all or most clinical trials. ^3-5^ Trial specific information includes the description of the study and what it will involve for a participant- including reasonably foreseeable, harm, discomfort, inconvenience and risk, and the benefits and alternatives to participating in the project. Generic information relevant to all trials includes confidentiality, compensation should injury occur, who to contact for more information or for concerns, that the trial is voluntary and the participant has the right to withdraw, and legal rights of subjects are not waived. National and international regulations have listed the essential components of an ICF (appendix 1) which are standard across trials. Ideally, the ICF should have a reading age of 11-12 years of age and be no longer than 4 pages long. ^6-10^

Research on informed consent has assessed comprehension, ^11-17^ readability, ^6-10,18^ and specific components of the ICF e.g. attitudes to certain phrases such as ‘randomisation’; as well as overall comprehension and recall of trial details. Findings from these studies have consistently demonstrated deficiencies in currently used ICFs. Comprehension is poor due to a number of factors: vocabulary is legalistic and scientific, there is a focus on mitigating risk rather than explaining the study itself, the ICF is too long and the readability is at a level consistent with a tertiary qualification. These factors are even more important when participants are unwell at the time consent is obtained: pain, anxiety, unwellness and lack of full comprehension of the medical condition may all affect the ability to understand and retain information.

A review of currently used ICFs in the Cardiovascular Research Unit has also identified limitations. In general ICF’s are too long (up to 26 pages long) and complex language is used. The section describing the trial itself has not increased significantly over time. However sections dealing with ‘confidentiality”, ‘voluntariness’ and ‘the rights to withdraw’ have become longer. The focus has thus shifted from informing participants to mitigating perceived risk. Sponsors often attempt to retain control of the ICF and lawyers add legalistic terms to the essential components of the ICF.

Long complex ICFs may be inappropriate for participant needs. ^2,19-23^ If the participant’s comprehension is less than optimal it is more difficult for them to make a reasoned, informed decision on their involvement in a trial.

One approach to improve informed consent in patients with acute medical problems is to initially use a short summary ICF, followed later by the standard long ICF. ^24,25^ While there is some improvement in comprehension, this process does not address some underlying problems. The long ICF is still used meaning participants have even more information to process, and the short ICF may not include all important information. Lack of standardisation allows sponsors to amend wording regarding participants rights, potentially abrogating some rights.

**Pilot study**

A pilot study was undertaken in CVRU this year to assess ICF’s and to see whether the new approach proposed here is viable. Ethics permission for the study was obtained in October 2010. All ICFs used at CVRU in the last 5 years (n=19) were assessed for length and readability. The average Flesch-Kincaid Grade level was 13.9, Flesch-Kincaid Reading Ease score: 29.9 and length was 15 pages long. ^25,26^ This implies that they were appropriate for a first year university student with high literacy skill. The most recent ICF’s (last 2 years) had an average length of 18 pages with the section on confidentiality increasing the most in length. The modified ICF and booklet have a reading age of 14 and have a readability score of 51.

27 patients in CCU and the cardiology ward were approached to assess the long ICF and questionnaire. 6 were excluded for the following reasons: poor literacy, ^5^ dementia, ^1^ frailty, ^1^ could not be bothered to read long document. ^1^ No English. ^1^ One patient was unexpectedly discharged and the questionnaire was not completed. The 20 who completed the study had an average score of 7.5/17 on the questionnaire with most having scores below 6, and 2 patients (with legal backgrounds) having very high scores of 15/17.

25 patients were approached to assess the short ICF, standardised booklet and questionnaire. All 25 completed the questionnaire and the average score was 11/17. This pilot study was used to assess the questionnaire and calculate sample size.

In summary current ICFs are long and difficult to read, and therefore do not facilitate the process of informed consent. A pilot study suggests that the proposed new approach to informed consent may improve the process.

# Aims of Research

## Aims

The aim of this study is to determine whether a modified ICF improves participants’ comprehension and recall of study information. Two modifications to the ICF will be evaluated; general information relevant to all clinical trials will be included in a separate standardised booklet, and trial specific information will be presented using simple English in a shorter ICF. The content validity of the booklet will also be assessed.

## Study hypotheses:

- The use of the study booklet alongside the unmodified trial specific ICF compared to the current ICFs will improve comprehension, particularly of patient rights are general aspects of trial design.
- The use of the study booklet and modified trial specific ICF (length shortened and readability improved) will further improve comprehension and recall.

## The objective

- to explore whether the use of a standardised booklet outlining information relevant to all clinical trials increases participants comprehension and recall of information about the trial.

*Content Validity*

To ensure the booklet includes all relevant general information pertaining to participant’s involvement in a trial, the validity of the booklet will be assessed prior to using it in the randomised trial. The face validity of the booklet has been established by showing it to current research participants (n=25), research nurses (N=4), investigators (n=6), independent research associates and monitors (n=5). The booklet has been found to be easily understood and to contain all essential components of consent that is not trial specific. Moreover the booklet has been found to address perceived deficiencies in current ICFs, i.e. trial terminology explained, specific sections for Maori.

Consultation with participants who have professional interest in the consent process will occur to ensure content validity prior to collating the version of the booklet to be used in the randomized trial.

*RCT to Evaluate Whether the Booklet Increases Participant Comprehension and Recall*

Once the content validity of the booklet has been established a randomised controlled trial will be undertaken to assess whether use of the standardised booklet improves patient recall and comprehension of a fictional trial compared with an existing ICF. Because the study booklet only focuses on general information relevant to all trials, the RCT will also evaluate whether reducing the length and increasing the readability of the trial specific ICF in addition to inclusion of the study booklet further increases patient recall and comprehension.

*Benefits of the Research*

The outcome of this research is to improve the informed consent process at ADHB, and elsewhere in New Zealand, by shifting the focus of informed consent from mitigating risks back to informing participants about the trial and their rights. The standard booklet should improve the consistency and standard of information given to participants regarding their rights.

This research aims to improve the informed consent process by shifting the focus of informed consent from mitigating risks to informing participants about the trial and their rights.

The use of an agreed standardised approach to providing general information relevant to all trials in the format of a booklet separate from trial specific information has the potential to:

- Reduce the use of legalistic jargon.
- Allow the trial specific ICF to focus on providing participants with relevant, trial specific information in a structured, concise way rather than have this information embedded in a lengthy document.
- Provide a consistent, nationally accepted approach endorsed by the New Zealand Health and Disability Ethics Committee to presenting general trial information that all studies could use.
- Improves the quality of the consent process by improving participant understanding.
- Allow inclusion of other information that may be useful to participants (e.g. definitions).
- Enable the inclusion of more complete information on the rights of special groups such as Maori.
- Ensure that changes to NZ law regarding rights are incorporated into the booklet without the need to gain ethics approval for every ICF for every study.
- Enable the booklet to be printed in different languages while maintaining consistency in the information included.
- Enable the ethics committees to focus on trial specific ICF’s, thereby reducing the workload related to reviewing long ICF’s.

# Research Design

## Evaluation of Booklet Validity

The face validity of the booklet has already been established (see above). 10-15 participants will be asked to assess the content validity of the booklet. The following individuals will be approached to review content validity.

Those who have already agreed to contribute are indicated*.

• *Mata Forbes and the MRCC

• A Pacific Island representative at ADHB and at least one other DHB;

• Representatives from the NZ Health and Disability Ethics Committees (including both lay and professional ethics committee members);

• *Representative from the ADHB legal team and at least one other DHB legal team;

• *Representatives from other DHBs;

• Vanya Kovach from University of Auckland Philosophy Department (previous chair of Northern X Ethics Committee with a research interest in applied and professional ethics);

• Representatives from the Health and Disability Commissioner;

• *Representatives from Clinical Research Organisations.

• *Ron Paterson Professor of Health Law and policy

• *Rosemary Deluca , Senior Lecturer Arts and Language Education, research interest in pedagogy of adult literacy in Ethics and informed consent

The above participants have been identified to ensure that consultation has taken place across a range of stakeholders who have professional interest in the consent process. It is expected that between 10-15 participants will be asked to assess the content validity of the booklet.

### Materials and Procedure

To assess the content validity of the booklet, each participant will be asked to rate whether the booklet addresses the eight pieces of general information relevant to all trials as specified by GCP. Each item will be rated on the following:

• How comprehensively each item is covered in the booklet on a three point scale (1 = not addressed; 2 = partially addressed but some information missing or incorrect; 3 = item fully addressed in booklet);

• How readable / understandable each of the items are presented in the booklet on a two point scale (1 = difficult to understand; 2 = easy to understand)

Participants will also be asked to rate overall whether the booklet meets the needs of GCP and the overall readability of the booklet using the scales outlined above and finally they will be asked to provide any suggestions or comments on the booklet. Participants will be contacted by phone or face to face to request their involvement in the study. Those who agree to participate will be sent by email an information sheet detailing the nature of the study and what their involvement will entail. They will also be sent a beta copy of the booklet (see appendix 2), the rating scale for assessing content validity and an outline of the GCP guidelines. Participants will be given one month to review the booklet and return their responses. Any non-responders will be followed up to ensure a reasonable response rate is achieved (minimum of 10 responses received).If following the review process there remain some issues or concerns about the validity of the booklet a meeting will be convened with a representative sample of the participants to discuss any concerns and identify a resolution.

## Randomised Study to Evaluate Consent Process Using Short ICF and Handbook Vs. Standard Consent

### Participants and Sample Size Calculations

282 stable patients at Auckland City Hospital will be randomly selected from all admissions to the cardiology services greater than 24 hours and who are expected to be in for a further 24 hours. To ensure a representative sample of ADHB patients admitted to the cardiology wards, the study will oversample for Maori and Pacific Island participants.

Potential participants will be excluded from the study if:

• They are a current trial participant;

• They have been involved in a clinical trial in the last two years;

• Are unable to read the ICF and complete the questionnaire unaided;

• Are unable to understand enough English to complete the questionnaires without help;

• Are medically unstable;

• Are confused as to the purpose of the study.

A screening database will be kept with a list of excluded / declined patients and reasons for their exclusion from the study. (Appendix 3) The database will be analysed to assess whether certain factors (e.g. education level) has lead to selection bias in this study.

Justification for sample size

This design is to test which variable (readability and length) affects recall and comprehension of the trial. It is estimated that the comprehension and recall score for those on the standard long ICF will average 7.5/17 and that of the short ICF and booklet will be 9.5/17. This is based on testing done on patients in the cardiology ward when developing the questionnaire and beta version of the booklet. A sample size of 60 completed patients in each group will have 80% power to detect a difference of 15% between the two groups, with an alpha level of 5%. In total 180 completers will thus be needed. To allow for 33% drop out for participants (unexpectedly discharged or transferred), the study will recruit 282 participants.

The pilot study undertaken over 2 weeks suggests that it will take 10 months to achieve the required sample size, based on the average number of patients admitted to the cardiology wards over 1 year and who are eligible to participate in the study.

### Materials

• The standard study ICF – All ICF’s approved by the ethics committee for the Cardiovascular Research Unit in the last 5 years has been reviewed and readability assessed using Flesch Reading Ease formula and the Flesch-Kincaid Grade Level. ^25,26^ A representative ICF will be chosen to be used in this study and adapted for the purposes of this study (e.g. the name of the sponsor, medication and laboratory will be amended to create a fictional ICF to reduce the risk of introducing a bias into the study). A fictitious ICF based on existing ICFs will be used to prevent any intellectual property concerns from sponsors about any current or previous trials and also to help eliminate any bias arising from limiting study participation to only a subsection of cardiology patients. The ICF will have a Flesch-Kincaid Grade level: 13.9, Flesch-Kincaid Reading Ease score: 29.9 and length of 15 pages.

• The short ICF with readability unchanged – The above standard study ICF will be amended with general trial information being removed from the ICF and instead the study booklet will be used. Trial specific information contained within the ICF will remain the same. There will be no change in the wording of the ICF once the general information is removed.

• The short ICF with readability changed – the trial specific information contained within the ICF will be amended so that it is no longer than 4-5 pages and will focus only on trial specific information. The reading age will be for an average 12-14 year old. 14,15

A questionnaire using multiple choice questions will be used to evaluate participants’ comprehension and recall (appendix 4). The questionnaire consists of the following:

• Study specific questions Q 1-8

• Standard information across trial Q9-13

Scoring of questionnaire

Based on the difficulty of the questions and the results of testing done on 26 participants, the questionnaire will be scored out of a total of 17 as follows:

Q 1-6, 10, 11, 13 – one point

Q 7-9 and 12- two points

### Procedure

Eligible participants will be approached and informed that the purpose of the study is to assess the consent process at Auckland City Hospital and that the trial described in the ICF is theoretical. They will be provided with an information sheet and consent form outlining the purpose of the study and what their participation will involve. It will be made clear that this is not an invitation into a clinical trial.

Participants who consent to participate will be randomised in 1:1:1 manner to one of the following study conditions using a computer algorithm to allocate patients to either arm of the study. The randomisation number will be noted in the questionnaire.

• C-ICF - long ICF which includes both trial specific and general trial information (current situation);

• Separated C-ICF – the ICF contains only the trial specific information (un-amended) and the study booklet;

• Amended C-ICF – the ICF contains only the trial specific information (amended to reduce length and improve readability) and study booklet.

Participants will be given at least 3-4 hours and up to of 24 hours to read the study documentation. This is in keeping with the ICH - GCP policy that participants must be given sufficient time to read the ICF. The questionnaire (appendix 4) will be then administered to assess comprehension and recall. It is preferable for the questionnaire to be filled in by the participant, and for it not to be verbally administered. The questionnaire will be checked to ensure that all questions answered. A simple assessment of ‘adult literacy in medicine’ (appendix 5) will also be administered.

## Analysis of Results

The primary analysis will be the total score out of 17 of the questionnaire in a quantitative manner with comparisons between the standard long consent and the short simple consent form with booklet process. A student t-test will be used to analyse the difference between the groups if normally distributed, and a Wilcoxon rank sum test will be used if distribution is not normal. ANOVA will be used to compare the likert scale results adjusted by significant covariates e.g. education level, first language, ethnicity and age. Baseline characteristics of participants will be analysed: age, education level, English as first language. Qualitative analyses will be done on questions requiring a narrative- e.g. the comments section.

Secondary analysis will evaluate predictors of poor understanding (e.g. education level, first language, ethnicity, age, adult literacy in medicine score) and their effects on comprehension and recall between the two ICFs.

## Significance of study

Informed consent is crucial to a successful trial. Participants are less likely to withdraw consent or be lost to follow up if they understand a trial. This study proposes a practical solution to potentially improve informed consent that can be used across the majority of studies. New Zealand is uniquely placed to implement results of this trial as it has centralised ethics committees and standardised indemnities for pharmaceutical trials, and ACC for investigator trials. A robust process to design and assess the booklet is required to convince ethics committees, sponsors and the Ministry of Health to enshrine this into research practice in New Zealand.

Initial feedback to this idea from a broad range of stakeholders including CRAs (contracted research organisations), pharmaceutical companies, investigators, study coordinators and ethics committee has been extremely positive. The consensus is that ICFs have become so complex that they have now become a barrier to consenting participants rather than a tool to explain a trial. The process does need to be evaluated in a trial to persuade international sponsors that this is a valid method to obtain consent.

# References

1 The National Commission for the Protection of Human Subjects of Biomedical and Behavioral Research. The Belmont Report: Ethical Principles and Guidelines for the protection of human subjects of research. http://ohsr od nih gov/guidelines/Belmont.html 1979.

2 Melby T, Mendelson JE, Jones RT. Patients' understanding of consent form should be checked before participation in trial. BMJ 1996; 312: 847.

3 World Medical Association. Declaration of Helsinki (1964). BMJ 1996; 313: 1448-9.

4 International Conference on Harmonisation Good Clinical Practice. International Conference on Harmonisation of technical requirements for registration of pharmaceuticals for human use: Informed consent of trial subjects [abstract]. http://ichgcp net/48-informed-consent-of-trial-subjects 2010.

5 US Food and Drug Administration. CFR - Code of Federal Regulations Title 21: Part 50 Protection of Human Subjects. http://www.accessdata.fda.gov/scripts/cdrh/cfdocs/cfcfr/CFRsearch cfm?CFRPart=50 2010.

6 Pfeffer N. How to get patients consent to enter clinical trials. Information and consent forms should use short words and sentences. BMJ 1996; 312: 186.

7 Goldfarb NM, DuBay WH. Writing good at a seventh grade reading level. Journal of Clinical Research Best Practices 2006; 2: 1-4.

8 Goldfarb NM. Measuring informed consent readability in Microsoft Word. Journal of Clinical Research Best Practices 2006; 2: 1-6.

9 Knapp P, Raynor DK, Silcock J, Parkinson B. Performance-based readability testing of participant materials for a phase I trial: TGN1412. J Med Ethics 2009; 35: 573-8.

10 Goldfarb NM. Readable informed consents are not optional. Journal of Clinical Research Best Practices 2005; 1: 1-7.

11 Joffe S, Cook EF, Cleary PD, Clark JW, Weeks JC. Quality of informed consent in cancer clinical trials: a cross-sectional survey. Lancet 2001; 358: 1772-7.

12 Howard JM, DeMets D. How informed is informed consent? The BHAT experience. Control Clin Trials 1981; 2: 287-303.

13 Bergler JH, Pennington AC, Metcalfe M, Freis ED. Informed consent: how much does the patient understand? Clin Pharmacol Ther 1980; 27: 435-40.

14 Miller C, Searight HR, Grable D, Schwartz R, Sowell C, Barbarash RA. Comprehension and recall of the informational content of the informed consent document: an evaluation of 168 patients in a controlled clinical trial. Journal of Clinical Research and Drug Development 1994; 8: 237-48.

15 Preziosi MP, Yam A, Ndiaye M, Simaga A, Simondon F, Wassilak SG. Practical experiences in obtaining informed consent for a vaccine trial in rural Africa. N Engl J Med 1997; 336: 370-3.

16 van Stuijvenberg M, Suur MH, de Vos S, Tjiang GC, Steyerberg EW, Derksen-Lubsen G, et al. Informed consent, parental awareness, and reasons for participating in a randomised controlled study. Arch Dis Child 1998; 79: 120-5.

17 Daugherty CK, Banik DM, Janish L, Ratain MJ. Quantitative analysis of ethical issues in phase I trials: a survey interview of 144 advanced cancer patients. IRB 2000; 22: 6-14.

18 Silva MC, Sorrell JM. Factors influencing comprehension of information for informed consent: ethical implications for nursing research. Int J Nurs Stud 1984; 21: 233-40.

19 Flory J, Emanuel E. Interventions to improve research participants' understanding in informed consent for research: a systematic review. JAMA 2004; 292: 1593-601.

20 Robinson EJ, Kerr CE, Stevens AJ, Lilford RJ, Braunholtz DA, Edwards SJ, et al. Lay public's understanding of equipoise and randomisation in randomised controlled trials. Health Technol Assess 2005; 9: 1-4.

21 Rogers CG, Tyson JE, Kennedy KA, Broyles RS, Hickman JF. Conventional consent with opting in versus simplified consent with opting out: an exploratory trial for studies that do not increase patient risk. J Pediatr 1998; 132: 606-11.

22 Williams BF, French JK, White HD, for the HERO-2 Consent Substudy Investigators. Informed consent during the clinical emergency of acute myocardial infarction (HERO-2 consent substudy): a prospective observational study. Lancet 2003; 361: 918-22.

23 Wirshing DA, Wirshing WC, Marder SR, Liberman RP, Mintz J. Informed consent: assessment of comprehension. Am J Psychiatry 1998; 155: 1508-11.

24 Agre P, Rapkin B. Improving informed consent: a comparison of four consent tools. IRB 2003; 25: 1-7.

25 Kincaid JP, Fishburne RP, Rogers RL, Chissom BS. Derivation of new readability formulas (Automated Readability Index, Fog Count and Flesch Reading Ease Formula) for Navy enlisted personnel. 1975. CNTECHTRA Research Branch Report. 8-75.

26 Kincaid JP, Braby R, Mears J. Electronic authoring and delivery of technical information. Journal of Instructional Development 1988; 11: 8-13.
